# Rock surfaces as reservoirs for airborne halophilic microorganisms in the Bochnia Salt Mine

**DOI:** 10.3389/fmicb.2026.1813537

**Published:** 2026-04-22

**Authors:** Magdalena Kowalewicz-Kulbat, Gabriela Arciszewska, Maciej Manecki, Dominika Drzewiecka, Michalina Rachubik, Karolina Stępniak, Luciana Albuquerque, Conceição Egas, Krzysztof Krawczyk, Camille Locht, Aleksandra Puławska

**Affiliations:** 1 Department of Immunology and Infectious Biology, Institute of Microbiology, Biotechnology and Immunology, Faculty of Biology and Environmental Protection, University of Lodz, Lodz, Poland; 2 Department of Mineralogy, Petrography and Geochemistry, Faculty of Geology, Geophysics and Environmental Protection, AGH University of Krakow, Krakow, Poland; 3 Department of Biology of Bacteria, Institute of Microbiology, Biotechnology and Immunology, Faculty of Biology and Environmental Protection, University of Lodz, Lodz, Poland; 4 CNC-UC – Center for Neuroscience and Cell Biology, University of Coimbra, Cantanhede, Portugal; 5 CIBB – Center for Innovative Biomedicine and Biotechnology, University of Coimbra, Cantanhede, Portugal; 6 Genoinseq – Next Generation Sequencing Unit, Cantanhede, Portugal; 7 Univ. Lille, CNRS, Inserm, CHU Lille, Institut Pasteur de Lille, U1019 – UMR9017 – CIIL – Center for Infection and Immunity of Lille, F-59000, Lille, France

**Keywords:** halite, halophilic archaea, hypersaline environments, microbial aerosolisation, rock-air interface, salt mine microbiology

## Abstract

Airborne halophilic archaea have recently been detected in subterranean salt mines, yet their origin in such dynamic environments remains unclear. We investigated whether exposed salt rock surfaces may serve as biological reservoirs for airborne halophilic microorganisms. Rock surfaces were sampled at three underground sites in the Bochnia Salt Mine (southern Poland), spanning a gradient of distance from the mine entrance and human influence. Surface swabs were collected from rock salt and salty claystone during summer and winter, representing contrasting warm-humid and cool-dry microclimatic conditions, and analyzed using cultivation-based methods combined with 16S rRNA gene sequencing. Across all sites, seasons, and both rock types considered together, halophilic microorganisms dominated rock-associated communities, occurring at approximately 1.2 × 10^3^ to 4.3 × 10^3^ CFU/25 cm^2^. In contrast, non-halophilic microorganisms were present at only about 0.5–1.3 × 10^2^ CFU/25 cm^2^. Mean halophilic abundance was approximately 3.8 × 10^3^ CFU/25 cm^2^ in summer and 1.4 × 10^3^ CFU/25 cm^2^ in winter, indicating less than two-fold seasonal variation on rock surfaces. In contrast, airborne communities from the same sites previously showed up to 13-fold summer-winter variation. A total of 11 halophilic archaeal species were identified on rock surfaces, dominated by members of the genus *Halococcus* (*Hcc. salifodinae*, *Hcc. hamelinensis*, *Hcc. morrhuae* and *Hcc. dombrowskii*), as well as *Natrinema versiforme* and *Halalkalicoccus paucihalophilus*. Most taxa detected on rock surfaces overlapped with those previously identified in the mine air. These results indicate that exposed salty rock surfaces constitute favorable habitats and likely persistent reservoirs for halophilic archaea, whereas the mine atmosphere appears to represent a transient, environmentally filtered compartment that receives only a subset of the rock-associated community.

## 1 Introduction

Salt rocks host diverse microbial communities, including halophilic microorganisms, that play an important role in geomicrobiological processes, long-term microbial survival and mineral–microbe interactions in hypersaline environments (Lowenstein et al., 2011; Jaakkola et al., 2016). Such communities are relevant not only for understanding the limits of life in evaporitic systems but also for applied contexts in which salt rocks constitute stable geological matrices, including underground resource storage and the preservation of biological signatures over geological timescales (Onstott et al., 2019; Dopffel et al., 2024). Beyond their geological and geomicrobiological relevance, subterranean salt mines are also widely used for therapeutic purposes based on inhalation of salt-saturated mine air, which has further motivated research into the composition, dynamics and origin of airborne microbial communities in these environments (Puławska et al., 2025).

Subterranean salt mines represent tightly coupled systems in which halite-rich rock surfaces, saline aerosols and mine air interact through ventilation and microclimatic gradients (Puławska et al., 2021a). Recent studies in the Bochnia Salt Mine demonstrated the presence of viable, cultivable halophilic archaea in mine air and revealed that their abundance and diversity are strongly modulated by distance from the mine entrance, relative humidity and seasonal changes (Puławska et al., 2025; Puławska et al., 2026). In contrast to non-halophilic microorganisms, whose occurrence in mine air can largely be attributed to external inputs or human activity, airborne halophilic archaea are evenly distributed across underground sites and largely absent at the mine entrance, suggesting an indigenous subterranean source (Puławska et al., 2025; Puławska et al., 2026).

The origin of these airborne halophilic archaea, however, has remained unresolved. Among potential reservoirs, salty rocks represent one of the most plausible persistent sources, as they provide long-term stability, high salinity, and protection from rapid environmental fluctuations (McGenity et al., 2001; Jaakkola et al., 2016). Seasonal changes in relative humidity are likely to influence the potential transfer of microorganisms from salt rock surfaces into the mine air via halite deliquescence and aerosol formation, whereas the persistence of rock-associated communities appears less sensitive to short-term atmospheric variability (Robinson et al., 2015; Puławska et al., 2026).

In this study, we tested the hypothesis that salt rock surfaces act as reservoirs supplying airborne halophilic microorganisms in the Bochnia Salt Mine. We compared the abundance and taxonomic composition of cultivable halophilic and non-halophilic microorganisms inhabiting the surfaces of two dominant rock types—rock salt (RS) and salty claystone (SC)—at three underground sites differing in distance from the mine entrance and human activity. Samples were collected during both summer and winter seasons, representing contrasting microclimatic regimes characterized by warm, humid summers and cool, relatively dry winters, and coinciding with previous airborne sampling campaigns. This design enabled direct comparison between rock-associated and airborne microbial communities under seasonally distinct environmental conditions. As in our previous studies (Puławska et al., 2025; Puławska et al., 2026) we focused here only on culturable microorganisms, as they may be valuable sources of novel compounds for biotechnological applications, including antimicrobials (Moopantakath et al., 2023) and anticancer compounds (Kowalewicz-Kulbat et al., 2023), which warrant further investigation.

## 2 Materials and methods

### 2.1 Sampling

Rock surfaces were sampled in June 2022 (summer season) and February 2023 (winter season) at three locations within the Bochnia Salt Mine, designated BL-2, BL-3, and BL-4 during the summer campaign and BZ-2, BZ-3, and BZ-4 during the winter campaign. Sites BL-3/BZ-3 (the Ważyn Chamber) are accessible to visitors, whereas sites BL-2/BZ-2 and BL-4/BZ-4 are not open to public access. Detailed descriptions of the mine layout, ventilation regime, microclimatic conditions and the exact spatial distribution of sampling sites are provided in our previous studies (Puławska et al., 2025; Puławska et al., 2026).

**Figure 1:**
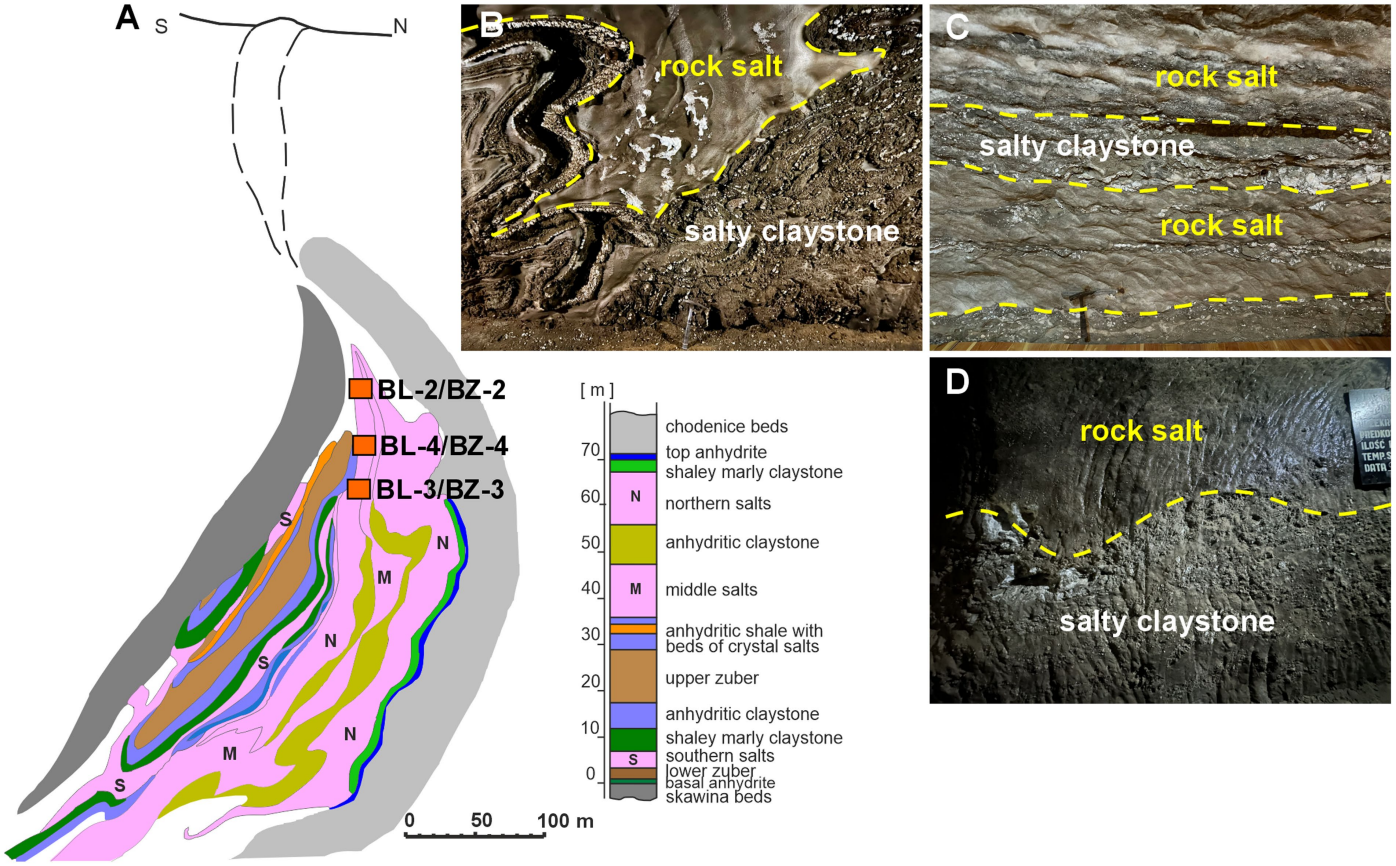
Geological setting of the Bochnia salt deposit and lithological context of sampled rock surfaces. **(A)** Simplified geological cross-section and lithostratigraphic profile of the Bochnia salt deposit showing the structure of the Miocene evaporite succession, modified from Puławska et al. (2021b) and originally based on Poborski (1952). **(B–D)** Representative photographs of underground mine walls illustrating the spatial association of rock salt (RS) and salty claystone (SC) at the sampling sites (BL-2/BZ-2, BL-3/BZ-3, and BL-4/BZ-4). Yellow dashed lines indicate lithological boundaries between halite-dominated layers and clay-rich evaporitic units.

At each site, samples were collected from two lithologically distinct rock types based on surface texture and mineralogical composition: smooth RS surfaces and rough SC surfaces. RS surfaces consisted predominantly of halite (NaCl) and formed compact, crystalline walls with smooth to slightly polished surfaces resulting from mining activity and prolonged exposure to mine air. These evaporitic rocks formed from the evaporation of Miocene seawater deposits, which formed approximately 13.6 million years ago (de Leeuw et al., 2010). In contrast, SC surfaces were rough and irregular and composed of fine-grained clastic sediments enriched with evaporitic minerals. They consisted mainly of clay minerals (including illite and kaolinite), quartz, halite, anhydrite, and gypsum, with additional contributions from carbonate minerals such as calcite and dolomite. These deposits originated from the accumulation of terrigenous material transported into a marine basin and subsequently mixed with evaporitic phases during sedimentation (Środoń, 1984; Garlicki, 2008). The regional geological setting, stratigraphy and evolution of the Bochnia Salt Mine within the Miocene evaporitic succession of the Carpathian Foredeep have been described in detail elsewhere and are therefore not discussed further here (e.g., Poborski, 1952; Garlicki, 1979). A geological cross-section of the deposit and the distribution of the different rock types at the sampling sites are shown in Figure 1.

The microbial samples were collected from the surface area limited by a square metal frame of 5x5cm (25 cm^2^) by scraping the entire surface using sterile collection swabs with a plastic stick and cotton tip (Medlab products, Raszyn, Poland) and immediately transferred into the 2 mL of liquid *Halobacterium* medium (HBM; 5 g/L yeast extract, 5 g/L casamino acids, 1 g/L Na-glutamate, 2 g/L KCl, 3 g/L Na_3_-citrate, 20 g/L MgSO_4_ x 7 H_2_O, 36 mg/L FeCl_2_ x 4 H_2_O, 360 ng/L MnCl_2_ x 4 H_2_O) containing 15, 20% or 25% NaCl and to a tube containing 0.85% NaCl. The samples were transferred from the salt mine to the laboratory within 8 h. At each sampling point samples were collected in triplicate.

### 2.2 Microbial isolation and culture conditions

One hundred μl of the swab samples in HBM media were plated onto solid HBM containing 20 g/L agar and 15, 20% or 25% NaCl and onto Tryptic-Soya Agar (TSA). The plates were incubated under the humid conditions at three temperatures (37 °C, 28 °C and 21 °C) up to 10 days for TSA plates and up to 3 months for HBM plates. After incubation the numbers of Colony Forming Units (CFU) were counted and expressed as CFU/25cm^2^ rock surface. Single colonies with red/reddish pigments growing on HBM agar were picked and cultured on fresh HBM agar with the same NaCl concentration as for the initial plates until the pure colonies were obtained. Similarly, colonies growing on TSA were picked and transferred to fresh TSA plates until pure colonies were obtained. The pure isolates were frozen in 50% glycerol and stored at −80 °C until further use.

### 2.3 Identification of halophilic microorganisms

Genomic DNA was extracted by using the Nielsen method (Nielsen et al., 1995). Briefly, the colonies from the HBM medium containing 15, 20 and 25% of NaCl were collected and suspended in TES buffer, centrifuged, re-suspended in the lysozyme solution (Merck) and incubated for 2 h in 37 °C. After incubation proteinase K was added and the samples were incubated for an additional 1.5 h at 37 °C. After addition of 7.5 M of ammonium acetate and incubation for 10 min on ice, the DNA was extracted by phenol:chloroform and isopropanol precipitation. The DNA pellet was then washed twice with 70% ethanol, air dried at room temperature, resuspended in 50-100 μL of the nuclease-free milliQ water and stored at −20° C. The 16S rRNA gene was amplified by PCR using primers 27F (5’-GAGTTTGATCCTGGCTCAG-3′) and 1525R (5’-AGAAAGGAGGTGATCCAGCC-3′) for bacteria and primers 21F (5’-TTCCGGTTGATCCTGCCGGA-3′) and 1492R (5’-TACGGYTACCTTGTTACG-3′) for archaea. The 16S rRNA PCR products were purified by using NZYGelpure (NZYtech) according to the manufacturer’s protocol and were stored at −20 °C until analyzed. The 16S rRNA gene sequence (~700 bp) was determined by Sanger sequencing (Stab Vida, Portugal) and used for the taxonomic affiliation of each isolate according to the online EzBioCloud database (Yoon et al., 2017). The DDBJ/ENA/GenBank accession numbers for the 16S rRNA gene sequence of strains BL28, BL31, BL35, BL36, BL37, BL38, BZ19, BZ34, BZ28, BZ39, BZ68, BZ1, BZ15, BZ125, BZ70, BZ6, BZ21, BZ27, BZ44, BZ56 and BZ66 are PZ221422, PZ221423, PZ221424, PZ221425, PZ221426, PZ221427, PZ221428, PZ221429, PZ221430, PZ221431, PZ221432, PZ221433, PZ221434, PZ221435, PZ221436, PZ221437, PZ221438, PZ221439, PZ221440, PZ221441 and PZ221442, respectively. For phylogenetic analysis, the 16S rRNA gene sequences were aligned with ClustalW (Thompson et al., 1994), and phylogenetic trees were reconstructed neighbor-joining (NJ) algorithm (Saitou and Nei, 1987) with the Jukes-Cantor model (Jukes and Cantor, 1969) using MEGA (version 11; Tamura et al., 2021). Bootstrap analysis based on 1000 replicates evaluated resulting tree topologies (Felsenstein, 1985).

### 2.4 Identification and biochemical analysis of non-halophilic microorganisms

Pure isolates of bacteria grown on TSA plates were identified using the VITEK 2 COMPACT system based on colorimetric reactions and analyzed using the Biomerieux database. This was outsourced to the diagnostic laboratory “Mikrografia” in Krakow. The bacteria were divided into three groups: non-sporulating GP (Gram-positive), BCL (Gram-positive spore-inducing bacilli), and GN (Gram-negative). In addition, the VITEK 2 system allowed us to characterize enzymatic activity, sugar metabolism and antibiotic resistance of the isolated bacteria.

### 2.5 Statistical analyses

Statistical analyses were performed with the GraphPad Prism 7 and STATISTICA 12.0 PL program. Data are expressed as means ± SD or medians ± interquartile range. Differences between samples were analyzed by the analysis of variance Kruskal-Wallis non-parametric test and Mann–Whitney U test. *p* values ≤0.05 were considered significant.

## 3 Results

### 3.1 Microbial sampling at different sites in the Bochnia Salt Mine

Microbial samples were taken in July 2022 and in February 2023 from three different sites of the Bochnia Salt Mine: BL-2/BZ-2, an underground tunnel, 1,185 m from the mine entry and 212 m below the ground surface; BL-3/BZ-3, the Wazyn chamber, 248 m deep and 1728 m from mine entry; and BL-4/BZ-4, 230 m deep and 2,671 m from mine entrance. These were the same sites as those described in our previous study and sampled for airborne microorganisms on the same days in the summer (Puławska et al., 2025) and winter (Puławska et al., 2026).

**Figure 2:**
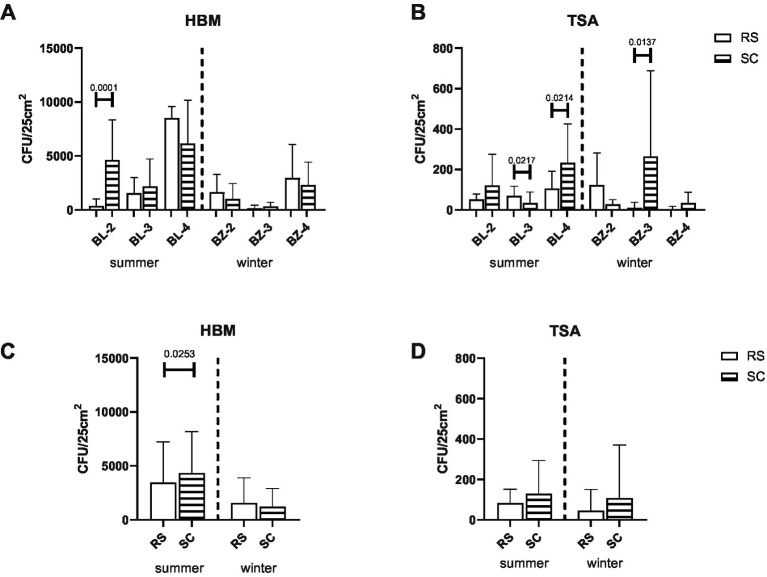
Presence of halophilic and non-halophilic microorganisms on the surface of rock salt (RS) and salty claystone (SC) at the three sampling sites. Colonies of halophilic and non-halophilic microorganisms collected from the swabs from the surface of the rocks at the indicated sampling sites in the summer and winter seasons and grown on HBM (panel **A**,**C**) or TSA (panel **B**,**D**) agar were counted after 3 or 1 month, respectively. The values shown in panel A represent the average CFU counts +/− standard deviations grown at HBM medium all salinities (15%, 20%, 25%) and all temperatures (room temperature 21 °C, 28 °C, 37 °C) together with three samples counted for each condition in each sampling sites in the summer time (BL-2–BL-4) and in the winter time (BZ-2–BZ-4). The values shown in panel B represent the average CFU counts +/− standard deviations grown at TSA medium at all temperatures together (21 °C, 28 °C, 37 °C) with three samples counted for each condition in each sampling sites in the summer time (BL-2–BL-4) and in the winter time (BZ-2–BZ-4). The values shown in panel C represent the average CFU counts +/− standard deviations grown at HBM medium all salinities (15, 20, 25%), all temperatures (21 °C, 28 °C, 37 °C), and all sampling sites (BL-2–BL-4) in the summer and (BZ-2–BZ-4) in the winter time, together with three samples counted for each condition. Values in panel D represent the average CFU counts +/− standard deviations grown on TSA agar all temperatures (21 °C, 28 °C, 37 °C) and all sampling sites (BL-2–BL-4) in the summer and (BZ-2–BZ-4) in the winter, together with three samples counted for each condition.

At each site, both RS and SC surfaces were sampled, as they represented the dominant lithologies exposed at the mine walls (Figure 1). 25 cm^2^ surfaces from each rock type of each sample site were swabbed and analyzed for the presence of halophilic and non-halophilic microorganisms grown on HBM containing either 15, 20% or 25% NaCl and TSA agar plates, respectively, at 21 °C, 28 °C or 37 °C. At each sampling sites, both the SC and RS surfaces were populated with live halophilic and non-halophilic microorganisms at roughly equal proportions between SC and RS surfaces (Figure 2). The most pronounced differences between SC and RS were found for the halophiles in BL-2 in the summer time (*p* < 0.0001; Figure 2A) and for the non-halophilic microorganisms in BZ-3 in the winter (*p* = 0.0137; Figure 2B), where the SC surfaces harbored more microorganisms than the RS surfaces. However, when all three sites were taken together, there was a significant difference between the number of halophiles present on the SC and RS surfaces in the summer (*p* = 0.0253; Figure 2C). No significance was seen between the number of halophiles present on the SC and RS surfaces in the winter (Figure 2C) and for non-halophilic microorganisms for the SC and RS surfaces in the winter and summer seasons (Figures 2C,D).

### 3.2 Microbial growth at different temperatures

**Figure 3:**
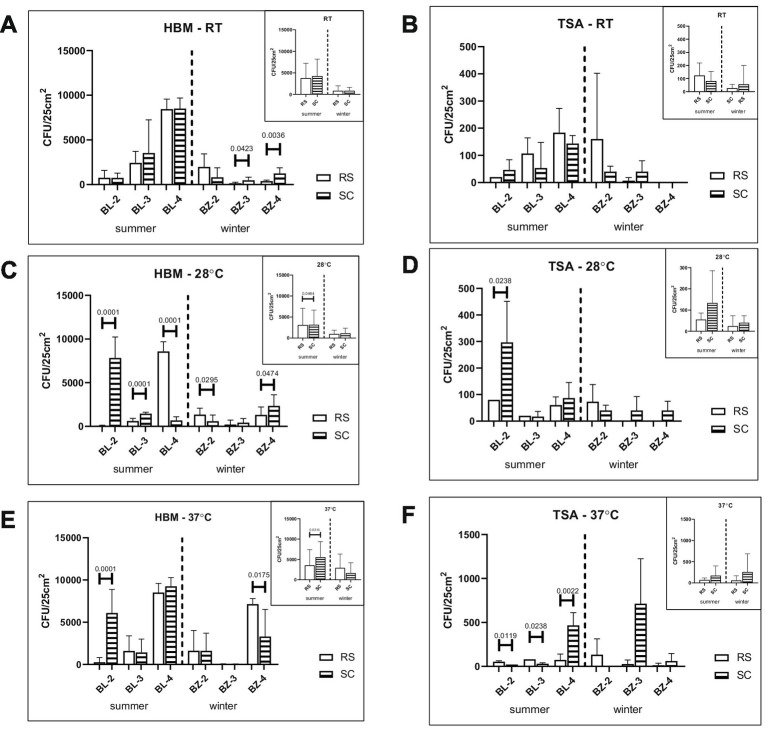
Presence of halophilic and non-halophilic microorganisms in the swabs from the rock salt (RS) and salty claystone (SC) at the three sampling sites in the summer and winter seasons. Colonies of halophilic microorganisms collected from the surface of RS and SC rocks at the indicated sampling sites and grown on HBM agar containing 15%, 20%, or 25% NaCl were counted after 3 months incubation at 21 °C, 28 °C, or 37 °C. The values shown in panel **(A)** represent the average CFU counts +/− standard deviations grown at all salinities at room temperature (RT; panel **(A)**), 28 °C (panel **(C)**), or 37 °C (panel **(E)**) with three samples counted for each condition. Values in panels **(B–F)** represent the average CFU counts +/− standard deviations of collected from the surface of RS and SC rocks at the indicated sampling sites non-halophilic microorganisms grown on TSA medium up to 10 days at room temperature (RT; panel **(B)**), 28 °C (panel **(D)**), or 37 °C (panel **(F)**) with three samples counted for each condition. Little squares incorporated in the upper right corner of the figure at each panel show the total numbers of colonies of halophilic and non-halophilic microorganisms grown on HBM (panel **(A,C,E)**) and TSA (panel **(B,D,F)**), respectively isolated from the surface of RS and SC rocks.

When the collected microbial communities were deconvoluted by growth temperature, no important differences between SC and RS surfaces were found for halophiles and non-halophilic microorganisms grown at room temperature (Figures 3A,B). Some differences between the two types of rock were found after growth at 28 °C and at 37 °C. In BL-2 SC surfaces contained more of either type of microorganisms than RS surfaces that grew at 28 °C (*p* < 0.0001 and *p* = 0.0238, Figures 3C,D, respectively), while in BL-4 the RS surface contained more halophiles than SC surface (*p* < 0.0001; Figure 3C). Significant differences between rock types were also found among microorganisms isolated in the summer that grew at 37 °C, especially in BL-2 for halophiles and BL4 for non-halophilic microorganisms (*p* < 0.0001 and *p* = 0.0022, Figures 3E,F, respectively), where SC surfaces contained more microorganisms than RS surfaces. However, when taken together for each temperature, differences were at best minor.

### 3.3 Halophile growth at different salinities

**Figure 4:**
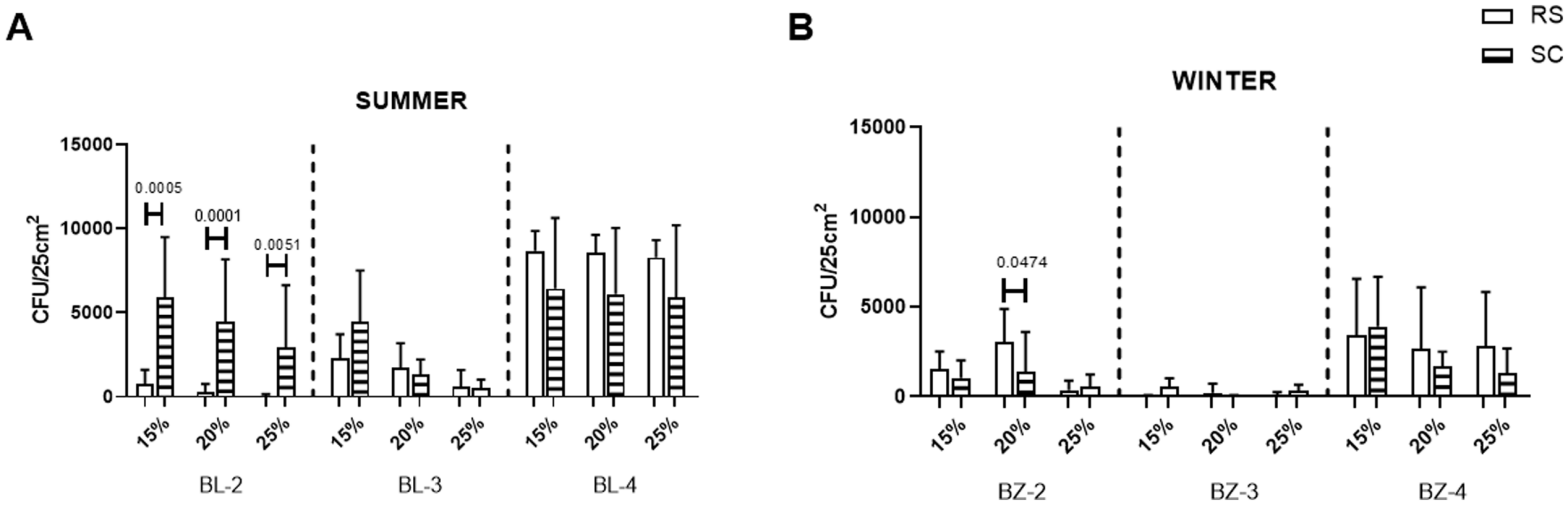
Presence of halophilic microorganisms isolated from the surface of RS and SC rocks in the summer season (panel **A**) and in the winter season (panel **B**) at the three sampling sites (BL-2 to BL-4) and (BZ-2 to BZ-4), respectively. HBM agar containing 15%, 20%, or 25% NaCl were counted after 3 months incubation at 21 °C, 28 °C, or 37 °C. The values shown represent the average CFU counts +/− standard deviations at all temperatures together, with three samples counted for each condition.

SC and RS surfaces were also compared for the presence of halophiles grown at different salinities. In the summer, in BL-2 almost all halophiles grown at either tested salinity came from the SC surfaces, while in BL-3 and BL-4 similar numbers of halophiles grown at either salinity were collected from both SC and RS surfaces (Figure 4A). Similarly, in the winter, no striking differences were seen between the numbers of halophiles collected from SC or RS surfaces of either sampling site after growth at either temperature (Figure 4B).

### 3.4 Comparison between the number of halophiles and non-halophilic microorganisms

**Figure 5:**
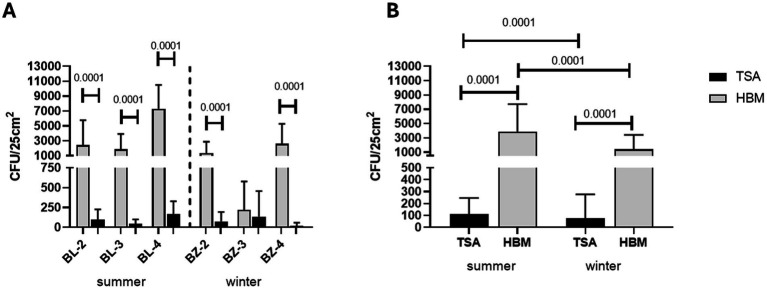
Total number of halophilic and non-halophilic microorganisms isolated from the surface of the rocks in the summer and winter seasons at the four sampling sites in the summer time (BL-2–BL-4) and in the winter time (BZ-2–BZ-4; panel **A**) or isolated from all sampling sites together (panel **B**). Colonies of halophilic microorganisms collected from the surface of the rocks at the indicated sampling sites and grown on HBM agar containing 15%, 20%, or 25% NaCl were counted after 3 months incubation at 21 °C, 28 °C, or 37 °C, while non-halophilic microorganisms were growing on TSA agar and counted after 10 days of incubation at 21 °C, 28 °C, or 37 °C. The values shown in panel **A** and **B** represent the average CFU counts +/− standard deviations with three samples counted for each condition.

When the amounts of halophiles and non-halophilic microorganisms collected from the rock surfaces of the three sampling sites were compared, up to more than a hundred-fold more halophiles were collected over non-halophilic microorganisms at almost each sampling site (*p* < 0.0001 for all differences). The exception was BZ-3, where roughly equal amounts of either microorganism were collected (Figure 5A). When taken together, in both the summer and the winter season, more than 10-times more live halophiles were collected from the rock surfaces than non-halophilic microorganisms (*p* < 0.0001; Figure 5B). When summer was compared to winter, significantly more microorganisms were collected from the rock surfaces in the summer than in the winter (*p* < 0.0001). However, these differences did not exceed a factor of two.

### 3.5 Species identification of halophilic microorganisms

The halophiles collected from the rock surfaces were mostly halophilic archaea. We therefore determined their species identity, found 11 different species (Table 1), established a similarity matrix (Supplementary Table S1) and constructed a phylogenetic tree (Supplementary Figure S1). Five species of archaea were isolated from both SC and RS surfaces, three only from the RS surfaces, and three only from the SC surfaces. Among the archaeal taxa detected, members of the *Halococcus* genus were dominant, including *Halococcus salifodinae*, *Halococcus hamelinensis*, *Halococcus morrhuae* and *Halococcus dombrowskii*, together with *Natrinema versiforme* and *Halalkalicoccus paucihalophilus*. *Haloarcula hispanica*, *Halococcus sedimicola* and *Halorubrum amylolyticum* were detected only on RS surfaces, whereas *Halococcus qingdaonensis* and *Haloarcula marismortui* were detected exclusively on SC surfaces. Although much less abundant than archaea, we could nevertheless identify 10 different halophilic bacterial species (Table 2), established a similarity matrix (Supplementary Table S2) and constructed a phylogenetic tree (Supplementary Figure S2). Four of them came from both types of rocks, while 3 only from the RS surfaces and 3 only from the SC surfaces. Among non-halophilic microorganisms, most were bacteria, and only very few live fungi were detected on the rock surfaces at either season. The bacteria were characterized by the VITEK 2 system (Supplementary Table S3), with a confidence level for all strains of >90% (except for *Lysinibacillus sphaericus*, for which the confidence level was 86%), but were not further studied here.

**Table 1:** Halophilic archeal species isolated from the rock surfaces of the Bochnia Salt Mine.

Halophilic species	RS^1^	SC^2^	Accession number	% identity^3^
*Halococcus salifodinae* DSM 8989^T^	+	+	AOME01000075	99.14
*Haloarcula hispanica* ATCC 33960^T^	+	−	CP002922	98.52
*Halococcus hamelinensis* 100A6^T^	+	+	AOMB01000011	99.76
*Halococcus qingdaonensis* CM5^T^	−	+	AY243109	99.68
*Halococcus morrhuae* DSM 1037^T^	+	+	AOMC01000054	99.48
*Haloarcula marismortui* ATCC 43049^T^	−	+	AY596298	99.19
*Halococcus sediminicola* CBA1101^T^	+	−	BBMP01000022	99.76
*Natrinema versiforme* XF10^T^	+	+	AB023426	98.65
*Halorubrum amylolyticum* ZC67^T^	+	−	KX376720	99.45
*Halalkalicoccus paucihalophilus* DSM 24557^T^	+	−	LTAZ01000003	99.22
*Halococcus dombrowskii* H4^T^	−	+	AJ420376	99.87

1 RS, rock salt.

2 SC, salty claystone.

3 Percent of 16S rRNA gene identity with the closest species with validly published names using the online EzBioCloud database (Yoon et al., 2017).

+, isolated, −, not isolated.

**Table 2:** Halophilic bacterial species isolated from the rock surfaces of the Bochnia Salt Mine.

Halophilic species	RS^1^	SC^2^	Accession number	% identity^3^
*Lentibacillus persicus* AMB31^T^	+	+	FN376846	99.90
*Chromohalobacter canadensis* ATCC 43984^T^	+	−	AJ295143	98.17
*Lentibacillus amyloliquefaciens* LAM0015^T^	+	+	CP013862	99.90
*Piscibacillus halophilus* HS224^T^	+	+	FM864227	99.69
*Virgibacillus kapii* KN3-8-4^T^	−	+	LC041942	100
*Alkalibacillus halophilus* YIM 012^T^	+	+	DQ359731	99.90
*Gracilibacillus thailandensis* TP2-8^T^	+	−	FJ182214	100
*Marinococcus halotolerans* NBRC 106070^T^	+	−	AB682353	99.74
*Chromohalobacter beijerinckii* ATCC 19372^T^	−	+	AB021386	98.59
*Salibacterium qingdaonense* CM1^T^	−	+	DQ115802	99.80

1 RS, rock salt.

2 SC, salty claystone.

3 Percent of 16S rRNA gene identity with the closest species with validly published names using the online EzBioCloud database (Yoon et al., 2017).

+, isolated, −, not isolated.

## 4 Discussion

### 4.1 Salt rock surfaces as microbial reservoirs in subterranean saline environments

In this study, we show that exposed salt rock surfaces in the underground Bochnia Salt Mine host abundant and diverse communities of viable halophilic microorganisms, dominated by halophilic archaea. Across all sampled sites and seasons, halophilic archaea were recovered from rock surfaces at approximately 100-fold higher densities than non-halophilic microorganisms, both in summer and winter.

This striking dominance contrasts sharply with airborne microbial communities from the same sites, where non-halophilic bacteria predominated during the winter season, except at the intermediate underground site BZ-2, which is distant from intensive human activity (Puławska et al., 2025; Puławska et al., 2026). A key difference between rock-associated and airborne halophilic communities lies in their seasonal dynamics. While airborne halophilic archaea exhibited pronounced seasonality, with densities up to 13-fold higher in summer than in winter (Puławska et al., 2026), seasonal differences on rock surfaces did not exceed a factor of two.

Although exposed rock surfaces do not provide the long-term environmental buffering characteristic of endolithic or subsurface habitats (Robinson et al., 2015; Meslier et al., 2018; Azua-Bustos et al., 2020; Casero et al., 2021), our results indicate that salt rock surfaces are a more biologically favorable habitat for halophilic archaea than the surrounding mine air. In the Bochnia Salt Mine, relative humidity ranged from approximately 65–77% in summer and 42–63% in winter, conditions that strongly modulate the persistence of saline aerosol microhabitats (Puławska et al., 2025; Puławska et al., 2026). Together, these observations support the interpretation that salt rocks function as spatially stable biological reservoirs for halophilic archaea, whereas the atmosphere appears to be a transient, environmentally filtered compartment that that may receive only a subset of the rock-associated community.

### 4.2 Salt rock surfaces as a geomicrobiological habitat at the lithosphere–atmosphere interface

Most studies investigating halophilic microorganisms associated with evaporitic rocks have focused on endolithic communities and typically rely on drilling, coring, or mechanical disruption of rock material to access microorganisms residing within the rock interior (e.g., Cycil et al., 2020; Megaw et al., 2019; Gramain et al., 2011; Pecher et al., 2020; Cockell et al., 2020; DasSarma et al., 2021; Schreder-Gomes et al., 2022). In contrast, the surface swabbing approach used in this study specifically targeted exposed rock surfaces and the rock–air interface, which remain comparatively underexplored despite their direct relevance to microbial aerosolization. By focusing on surface-associated communities, our data provide complementary insight into the ecological link between halite-rich substrates and airborne halophilic microorganisms in subterranean saline environments.

Rock surfaces in subterranean salt mines should be regarded as active geomicrobiological habitats rather than inert substrates. Numerous studies have demonstrated that exposed rock surfaces host structured subaerial biofilms (SABs) that occupy the dynamic interface between the lithosphere and the atmosphere, where microbial survival is governed by mineral composition, surface roughness, porosity and microtopography, as well as by fluctuations in humidity and temperature (Gorbushina, 2007; Gorbushina and Broughton, 2009; Ronan et al., 2013; Uroz et al., 2015; Stone et al., 2016; Cuadros, 2017; Meslier et al., 2018; Wierzchos et al., 2018; Fomina and Skorochod, 2020; Casero et al., 2021). These biofilms are highly organized microbial systems that persist under extreme physicochemical stress, including desiccation, nutrient limitation, and high salinity (Azua-Bustos et al., 2020; Dopffel et al., 2021; Medina-Chávez et al., 2023). In saline environments, such as salt mines, these interactions are further modulated by the hygroscopic properties of halite and associated evaporitic minerals, which promote capillary condensation and episodic deliquescence, generating microscale aqueous niches even when bulk air humidity remains below saturation (Davila et al., 2008; Wierzchos et al., 2012; Robinson et al., 2015; Puławska et al., 2021a).

Although RS and SC differ in mineralogical composition and surface structure, both rock types contain substantial amounts of halite and provide physicochemical microenvironments that retain moisture through hygroscopicity. Such microscale brine films and associated biofilms are known to protect microbial cells against rapid desiccation and oxidative stress (Davila et al., 2008; Casero et al., 2021; Gorbushina and Broughton, 2009). While surface habitats remain linked to atmospheric humidity, the hygroscopic nature of the substrate likely attenuates the impact of short-term fluctuations compared to the airborne state, supporting the persistence of rock-associated halophilic communities (Wierzchos et al., 2012; Azua-Bustos et al., 2020). Importantly, this rock-surface-associated mode of persistence contrasts with the transient nature of the mine atmosphere, where microbial survival is strongly constrained by humidity-dependent aerosol formation. While elevated relative humidity promotes halite deliquescence and facilitates the aerosolization and dispersal of halophilic microorganisms into the air (Puławska et al., 2026), the rock surface itself represents a far more stable physicochemical compartment. In addition to deliquescence and salt hygroscopy, physical processes may also contribute to the mobilization of microorganisms from rock surfaces into the mine air. Previous aerosol studies in the Bochnia Salt Mine identified geogenic airborne particles derived from weathering and abrasion of rock surfaces, indicating that physical mobilization of salt-derived material occurs in this environment (Puławska et al., 2021b; Puławska et al., 2021a). Ventilation-driven airflow and local turbulence may therefore facilitate the detachment of cell-bearing salt microfragments from moist surface crusts. In contrast, human activity appears to be associated mainly with transient inputs of non-halophilic microorganisms (Puławska et al., 2025; Puławska et al., 2026). In this context, salt rocks function not merely as passive substrates but as long-term microbial reservoirs that sustain halophilic populations and may supply cells to the atmosphere when microclimatic conditions become favorable. This conceptual separation between rocks as stable source habitats and air as a dynamic, environmentally filtered compartment provides a plausible ecological framework linking lithic colonization to the observed patterns of airborne halophilic archaea in the Bochnia Salt Mine.

### 4.3 Taxonomic continuity between rock-associated and airborne communities

**Figure 6:**
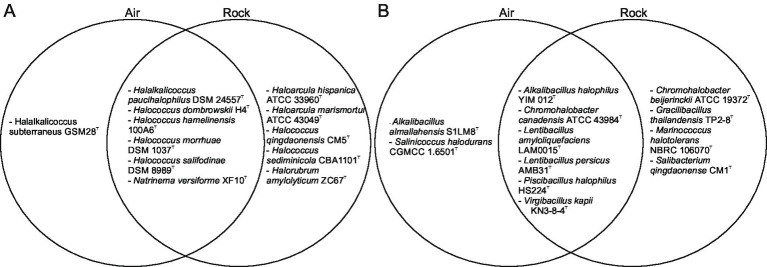
Venn diagram showing the presence of halophilic archaea **(A)** and bacteria **(B)** detected in the air and/or rock surfaces, as indicated.

The taxonomic composition of halophilic archaea isolated from rock provides qualitative support for the hypothesis that salt rocks represent an important reservoir, and a likely source habitat, for airborne halophiles in the Bochnia Salt Mine. A total of 11 halophilic archaeal species were identified on rock surfaces, 6 of which, mainly representatives of the genus *Halococcus* (*Hcc. salifodinae*, *Hcc. hamelinensis*, *Hcc. morrhuae*, and *Hcc. dombrowskii*), as well as *Nnm. versiforme* and *Hac. paucihalophilus*, had previously been detected in airborne samples from the mine (Puławska et al., 2025; Puławska et al., 2026). This extensive overlap (Figure 6A) is consistent with the view that the airborne halophilic community may largely represent a subset of the more diverse rock-associated assemblage.

At the same time, several halophilic archaeal species identified on rock surfaces were absent from airborne samples, suggesting selective constraints on aerosolization and atmospheric persistence. Such asymmetry is consistent with the view that the mine atmosphere acts as an ecological filter, allowing only a fraction of the rock-resident community to enter and survive in the aerial compartment. Similar habitat-specific partitioning has been described for halophilic archaea inhabiting halite and other evaporitic substrates, where endolithic or surface-associated populations develop distinct community structures compared with those found in surrounding environments, such as soils (Robinson et al., 2015; Azua-Bustos et al., 2020).

Notably, the dominance of *Halococcus* species among both rock-associated and airborne halophiles is ecologically coherent, as members of this genus are well known for their exceptional resistance to desiccation and prolonged nutrient limitation thanks to their rigid polysaccharide cell walls, traits that favor survival at the rock–air interface and during atmospheric transport (Stan-Lotter et al., 1999; Fendrihan et al., 2009; Oren, 2012). In addition, *Halococcus* species exhibit pronounced resistance to oxidative stress and radiation, as demonstrated under simulated space and extraterrestrial conditions (Leuko et al., 2020), further supporting their capacity to persist during aerosolisation and airborne dispersal.

In addition to halophilic archaea, halophilic bacteria also exhibited pronounced taxonomic continuity between rock-associated and airborne communities. Among the 10 halophilic bacterial species identified on rock surfaces, six were likewise detected in airborne samples (Figure 6B), indicating that halophilic bacteria, together with archaea, may participate in microbial connectivity between rock surfaces and air within the mine system. In particular, taxa such as *Marinococcus halotolerans*, *Gracilibacillus thailandensis*, *Chromohalobacter beijerinckii* and *Salibacterium qingdaonense* were restricted to rock surfaces, whereas other halophilic bacterial species were shared between rock-associated and airborne communities.

Despite species-level differences between rock-associated and airborne communities, the overall abundance of halophiles did not differ substantially between RS and SC surfaces. While RS and SC differ in mineralogical composition and surface microstructure, RS forming relatively smooth crystalline surfaces and SC exhibiting higher roughness and microtopographic heterogeneity, these differences did not translate into major quantitative contrasts in halophilic microbiota abundance. This suggests that, under the saline underground conditions of the Bochnia Salt Mine, the presence of halite and its hygroscopic behavior appear to represent dominant environmental features associated with the persistence of halophilic microbiota across different rock types.

Nevertheless, the occurrence of certain archaeal taxa exclusively on RS or SC surfaces suggests that lithological and microstructural heterogeneity may shape community composition at the species level. In this context, *Har. hispanica*, *Hcc. sedimicola* and *Hrr. amylolyticum*, which were recovered only from RS surfaces, may be adapted to chemically more homogeneous but highly saline substrates, whereas *Hcc. qingdaonensis* and *Har. marismortui*, detected exclusively on SC surfaces, may benefit from access to a more mineralogically diverse matrix. Such lithology-associated effects appear secondary to halite availability and do not override the likely importance of rock surfaces as long-term reservoirs of halophilic archaea potentially contributing to airborne populations under favorable microclimatic conditions.

### 4.4 Limitations

While our results provide convergent quantitative and qualitative evidence consistent with the hypothesis that salt rock surfaces act as important reservoirs for airborne halophilic archaea in the Bochnia Salt Mine, several limitations of the present study should be acknowledged. In particular, our analyses were restricted to culturable microorganisms inhabiting exposed rock surfaces and did not address non-culturable taxa or deeper endolithic communities within the rock matrix. Accordingly, the present study does not capture the full microbial diversity associated with the rock surfaces or the mine atmosphere, and the conclusions should be interpreted as referring specifically to the culturable fraction. Moreover, although surface swabbing enabled direct investigation of the rock–air interface most relevant for aerosolization, it does not resolve whether the detected microorganisms represent actively growing populations or long-term dormant assemblages intermittently reactivated by microclimatic changes. Moreover, the observed taxonomic overlap between rock-associated and airborne communities supports, but does not directly demonstrate, the proposed role of rock surfaces as contributors to airborne halophilic microorganisms. These considerations highlight important avenues for future research and frame the scope of the conclusions drawn here.

### 4.5 Concluding remarks

In summary, this study indicates that exposed salt rock surfaces in the Bochnia Salt Mine constitute stable, long-term reservoirs of viable culturable halophilic archaea, whereas the mine atmosphere represents a transient and environmentally filtered compartment. By combining surface-based cultivation with seasonal sampling, we show that airborne halophilic communities overlap substantially with rock-associated populations and are consistent with the hypothesis that rock surfaces contribute to airborne communities under favorable microclimatic conditions. These findings provide a plausible ecological framework linking lithic microbial persistence to aerosolized microbiota in subterranean salt environments. Beyond their ecological significance, our results highlight the importance of integrating geomicrobiology with microclimatic monitoring to better understand microbial dynamics in salt mines used for recreational and therapeutic purposes.

## Data Availability

The datasets presented in this study can be found in online repositories. The names of the repository/repositories and accession number(s) can be found in the article/Supplementary material.
