# Supplementary material for: Rock surfaces as reservoirs for airborne halophilic microorganisms in the Bochnia Salt Mine
Source: Front Microbiol. 2026 Apr 22;17:1813537. doi: 10.3389/fmicb.2026.1813537 (PMC13144014; doi:10.3389/fmicb.2026.1813537)
Supplement: Supplementary file 1 [file Data_Sheet_1.PDF]

Figure S1

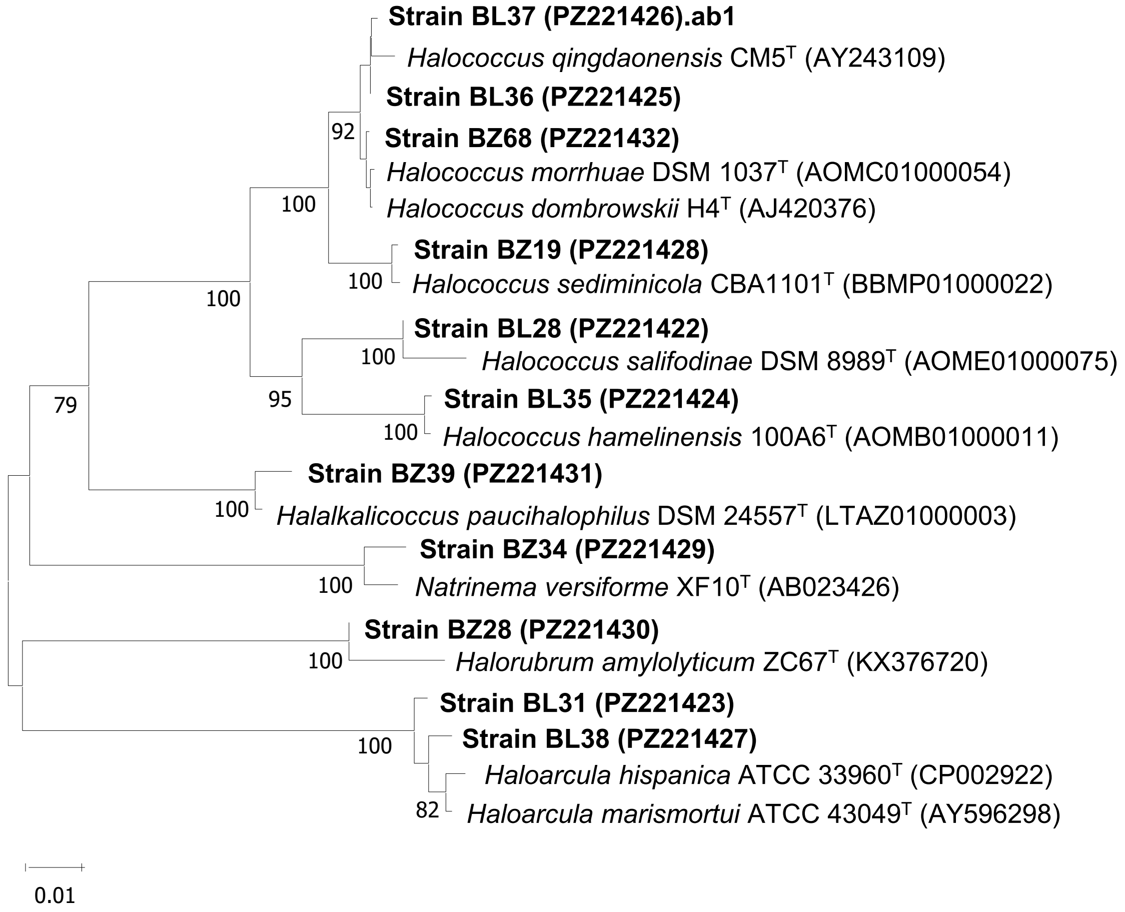

Figure S1: Phylogenetic dendogram based on 16S rRNA gene sequences showing the relationship of halophilic archael isolates (in bold) and the closest described species (accession numbers between brackets). The phylogenetic relationships were reconstructed using the MEGA 11 software package and the NJ method. Bootstrap values (%) derived from 1000 replications are shown for branches with more than 70% bootstrap support. Scale bar, 1 inferred nucleotide substitutions per 100 nucleotides.

Figure S2

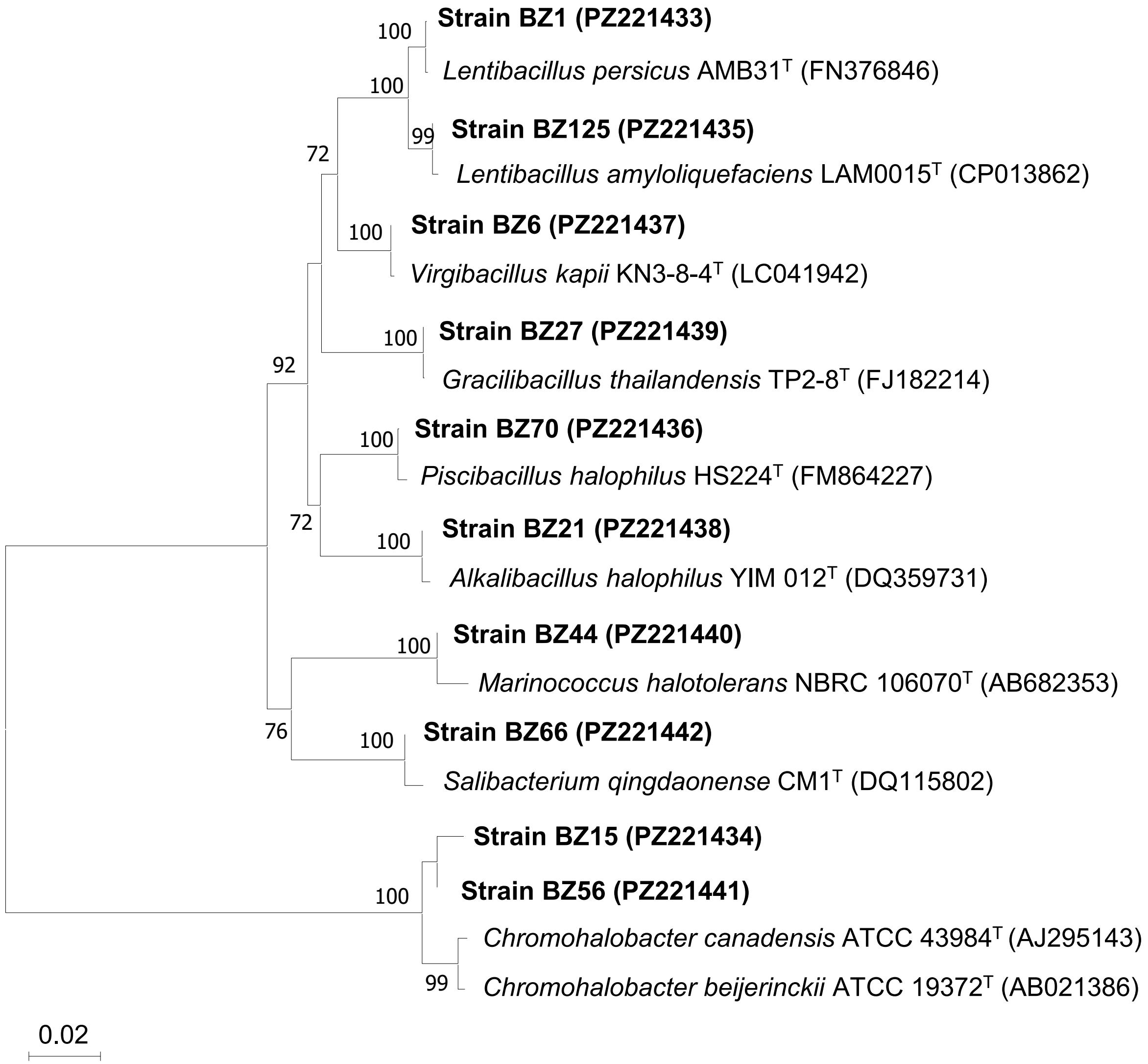

Figure S2: Phylogenetic dendogram based on 16S rRNA gene sequences showing the relationship of halophilic bacterial isolates and the closest described species. The phylogenetic relationships were reconstructed using the MEGA 11 software package and the NJ method. Bootstrap values (%) derived from 1000 replications are shown for branches with more than 70% bootstrap support. Scale bar, 2 inferred nucleotide substitutions per 100 nucleotides
