# Supplementary material for: Rock surfaces as reservoirs for airborne halophilic microorganisms in the Bochnia Salt Mine
Source: Front Microbiol. 2026 Apr 22;17:1813537. doi: 10.3389/fmicb.2026.1813537 (PMC13144014; doi:10.3389/fmicb.2026.1813537)
Supplement: Supplementary file 4 [file Table_3.DOCX]

|  | | **GP** | | | | | | | | | | | |
| --- | --- | --- | --- | --- | --- | --- | --- | --- | --- | --- | --- | --- | --- |
|  |  | **Genus** | *Kocuria* | | | *Micrococcus* | | | | | | *Staphylococcus* | *Dermacoccus* |
|  |  | **Species** | *rosea* | *rhizophila* | *kristinae* | *luteus* | | | | | | *epidermidis* | *nishinomiyaensis* |
|  |  | **Sample** | 25D | 28D | 34D | 21D | 27D | 38D | | 40D | | 29D | 35D |
| **SUGAR BREAKDOWN** | D-AMYGDALIN | ***AMY*** | **-** | **-** | **-** | **-** | **-** | **-** | | **-** | | **-** | **-** |
|  | D-XYLOSE | ***dXYL*** | **-** | **-** | **-** | **-** | **-** | **-** | | **-** | | **-** | **-** |
|  | CYCLODEXTRIN | ***CDEX*** | **-** | **-** | **-** | **-** | **-** | **-** | | **-** | | **-** | **-** |
|  | D-SORBITOL | ***dSOR*** | **-** | **-** | **-** | **-** | **-** | **-** | | **-** | | **-** | **-** |
|  | D-GALACTOSE | ***dGAL*** | **-** | **-** | **-** | **-** | **-** | **-** | | **-** | | **+** | **-** |
|  | D-RIBOSE | ***dRIB*** | **-** | **-** | **-** | **-** | **-** | **-** | | **-** | | **-** | **-** |
|  | LACTOSE | ***LAC*** | **-** | **-** | **-** | **-** | **-** | **-** | | **-** | | **+** | **-** |
|  | N-ACETYL-D-GLUCOSAMINE | ***NAG*** | **-** | **-** | **-** | **-** | **-** | **-** | | **-** | | **-** | **-** |
|  | D-MALTOSE | ***dMAL*** | **-** | **-** | **-** | **-** | **-** | **-** | | **-** | | **+** | **-** |
|  | D-MANNITOL | ***dMAN*** | **-** | **-** | **-** | **-** | **-** | **-** | | **-** | | **-** | **-** |
|  | D-MANNOSE | ***dMNE*** | **-** | **-** | **-** | **-** | **-** | **-** | | **-** | | **+** | **-** |
|  | METHYL-B-D-GLUCOPYRANOSIDE | ***MBdG*** | **-** | **-** | **-** | **-** | **-** | **-** | | **-** | | **-** | **-** |
|  | PULLULAN | ***PUL*** | **-** | **-** | **-** | **-** | **-** | **-** | | **-** | | **-** | **-** |
|  | D-RAFFINOSE | ***dRAF*** | **-** | **-** | **-** | **-** | **-** | **-** | | **-** | | **-** | **-** |
|  | SACCHAROSE/SUCROSE | ***SAC*** | **-** | **-** | **-** | **-** | **-** | **-** | | **-** | | **-** | **-** |
|  | D-TREHALOSE | ***dTRE*** | **-** | **-** | **-** | **-** | **-** | **-** | | **-** | | **-** | **-** |
| **ENZYMATIC ACTIVITY** | PHOSPHATIDYLINOSITOL PHOSPHOLIPASE | ***CPIPLC*** | **-** | **-** | **-** | **-** | **-** | **-** | | **-** | | **-** | **-** |
|  | ARGININE DIHYDROLASE 1 | ***ADH1*** | **+** | **+** | **-** | **-** | **+** | **-** | | **-** | | **+** | **-** |
|  | BETA-GALACTOSIDASE | ***BGAL*** | **-** | **-** | **+** | **-** | **-** | **-** | | **-** | | **+** | **-** |
|  | ALPHA-GLUCOSIDASE | ***AGLU*** | **-** | **-** | **-** | **+** | **+** | **+** | | **+** | | **+** | **+** |
|  | ALA-PHE-PRO ARYLAMIDASE | ***APPA*** | **+** | **-** | **-** | **-** | **+** | **+** | | **+** | | **-** | **+** |
|  | L-ASPA21ATE ARYLAMIDASE | ***AspA*** | **-** | **-** | **-** | **-** | **-** | **-** | | **-** | | **-** | **-** |
|  | BETA GALACTOPYRANOSIDASE | ***BGAR*** | **-** | **-** | **-** | **-** | **-** | **-** | | **-** | | **-** | **-** |
|  | ALPHA-MANNOSIDASE | ***AMAN*** | **-** | **-** | **-** | **-** | **-** | **-** | | **-** | | **-** | **-** |
|  | PHOSPHATASE | ***PHOS*** | **-** | **-** | **-** | **-** | **-** | **-** | | **-** | | **+** | **-** |
|  | LEUCINE ARYLAMIDASE | ***LeuA*** | **+** | **-** | **-** | **+** | **+** | **+** | | **+** | | **-** | **+** |
|  | L-PROLINE ARYLAMIDASE | ***ProA*** | **-** | **-** | **+** | **+** | **+** | **+** | | **+** | | **-** | **-** |
|  | BETA GLUCURONIDASE | ***BGURr*** | **-** | **-** | **-** | **-** | **-** | **-** | | **-** | | **-** | **-** |
|  | ALPHA-GALACTOSIDASE | ***AGAL*** | **-** | **-** | **-** | **-** | **-** | **-** | | **-** | | **-** | **-** |
|  | L-PYRROLYDONYL-ARYLAMIDASE | ***PyrA*** | **-** | **+** | **-** | **+** | **-** | **+** | | **+** | | **-** | **-** |
|  | BETA-GLUCURONIDASE | ***BGUR*** | **-** | **-** | **-** | **-** | **-** | **-** | | **-** | | **-** | **-** |
|  | ALANINE ARYLAMIDASE | ***AlaA*** | **+** | **+** | **+** | **+** | **-** | **-** | | **-** | | **-** | **+** |
|  | TYROSINE ARYLAMIDASE | ***TyrA*** | **-** | **-** | **-** | **-** | **+** | **+** | | **-** | | **-** | **-** |
|  | UREASE | ***URE*** | **-** | **-** | **-** | **+** | **-** | **-** | | **-** | | **+** | **-** |
|  | SALICIN | ***SAL*** | **-** | **-** | **-** | **-** | **-** | **-** | | **-** | | **-** | **-** |
|  | ARGININE DIHYDROLASE 2 | ***ADH2s*** | **-** | **-** | **-** | **-** | **-** | **-** | | **-** | | **+** | **-** |
| **ANTIBIOTIC RESISTANCE** | POLYMIXIN B RESISTANCE | **POLYB** | **-** | **-** | **-** | **-** | **-** | | **-** | | **-** | **-** | **-** |
|  | BACITRACIN RESISTANCE | **BACI** | **-** | **-** | **-** | **-** | **-** | | **-** | | **-** | **+** | **-** |
|  | NOVOBIOCIN RESISTANCE | ***NOVO*** | **-** | **-** | **-** | **-** | **-** | | **-** | | **-** | **-** | **-** |
|  | O/129 RESISTANCE (COMP.VIBRIO.) | ***O129R*** | **-** | **-** | **-** | **-** | **-** | | **-** | | **-** | **+** | **-** |
|  | OPTOCHIN RESISTANCE | ***OPTO*** | **-** | **-** | **-** | **-** | **-** | | **-** | | **-** | **+** | **-** |
| **OTHER** | L-LACTATE ALKALINIZATION | ***ILATk*** | **-** | **+** | **-** | **+** | **+** | | **-** | | **+** | **+** | **-** |
|  | GROWTH IN 6.5% NACL | ***NC6.5*** | **-** | **-** | **+** | **-** | **-** | | **-** | | **-** | **+** | **-** |

**Supplementary Table S3:** Biochemical characteristics of Gram-positive (GP), Gram-positive spore-forming bacilli (BCL), and Gram-negative (GN) microorganisms isolated from the rocks of Bochnia Salt Mine, identified by VITEK-2 system

|  | | **BCL** | | | | | | | | | | |
| --- | --- | --- | --- | --- | --- | --- | --- | --- | --- | --- | --- | --- |
|  |  | **Genus** | *Bacillus* | | | *Aneurinibacillus* | *Lysinibacillus* | *Sphingomonas* | *Brevibacillus* | *Unidentified* | | |
|  |  | **Species** | *smithii* | *clausii* | | *aneurinilyticus* | *sphaericus* | *paucimobilis* | *borstelensis* | *nd* | *nd* | *nd* |
|  |  | **Sample** | 30D | 36D | 37D | 23D | 32D | 33D | 39D | 22D | 26D | 31D |
| **SUGAR BREAKDOWN** | D-GALACTOSE | ***DGAL*** | **-** | **-** | **-** | **-** | **-** | **-** | **-** | **-** | **-** | **-** |
|  | GLYCOGEN | ***GLYG*** | **-** | **-** | **-** | **-** | **-** | **-** | **-** | **-** | **-** | **-** |
|  | MYO-INOSITOL | ***INO*** | **-** | **-** | **-** | **-** | **-** | **-** | **-** | **-** | **-** | **-** |
|  | METHYL-D-XYLOSIDE | ***MDX*** | **-** | **-** | **-** | **-** | **-** | **-** | **-** | **-** | **-** | **-** |
|  | MALTOTRIOSE | ***MTE*** | **-** | **-** | **-** | **-** | **-** | **-** | **-** | **-** | **-** | **-** |
|  | D-MANNITOL | ***DMAN*** | **-** | **-** | **-** | **-** | **-** | **-** | **-** | **-** | **-** | **-** |
|  | D-MANNOSE | ***DMNE*** | **-** | **-** | **-** | **-** | **-** | **-** | **-** | **-** | **-** | **-** |
|  | D-MELEZITOSE | ***DMLZ*** | **-** | **-** | **-** | **-** | **-** | **-** | **-** | **-** | **-** | **-** |
|  | PALATINOSE | ***PLE*** | **-** | **-** | **-** | **-** | **-** | **-** | **-** | **-** | **-** | **-** |
|  | L-RHAMNOSE | ***IRHA*** | **-** | **-** | **-** | **-** | **-** | **-** | **-** | **-** | **-** | **-** |
|  | PYRUVATE | ***PVATE*** | **-** | **-** | **-** | **-** | **-** | **-** | **-** | **-** | **-** | **-** |
|  | D-TAGATOSE | ***DTAG*** | **-** | **-** | **-** | **-** | **-** | **-** | **-** | **-** | **-** | **-** |
|  | D-TREHALOSE | ***DTRE*** | **-** | **-** | **-** | **-** | **-** | **-** | **-** | **-** | **-** | **-** |
|  | INULIN | ***INU*** | **-** | **-** | **-** | **-** | **-** | **-** | **-** | **-** | **-** | **+** |
|  | D-GLUCOSE | ***DGLU*** | **-** | **-** | **-** | **-** | **-** | **-** | **-** | **-** | **-** | **-** |
|  | D-RIBOSE | ***DRIB*** | **-** | **-** | **-** | **-** | **-** | **-** | **-** | **-** | **-** | **-** |
| **ENZYMATIC ACTIVITY** | BETA-XYLOSIDASE | ***BXYL*** | **-** | **+** | **+** | **-** | **+** | **+** |  | **+** | **+** | **+** |
|  | L-LYSINE-ARYLAMIDASE | ***LYSA*** | **-** | **+** | **+** | **-** | **-** | **-** | **-** | **+** | **-** | **-** |
|  | L-ASPA21ATE ARYLAMIDASE | ***ASPA*** | **-** | **-** | **+** | **-** | **-** | **-** | **-** | **+** | **-** | **-** |
|  | EUCINE-ARYLAMIDASE | ***LEUA*** | **-** | **+** | **+** | **-** | **+** | **+** | **+** | **+** | **-** | **-** |
|  | PHENYLALANINE ARYLAMIDASE | ***PHEA*** | **-** | **+** | **+** | **+** | **+** | **+** | **+** | **+** | **+** | **+** |
|  | L-PROLINE ARYLAMIDASE | ***PROA*** | **-** | **-** | **-** | **-** | **-** | **-** | **-** | **-** | **-** | **-** |
|  | BETA-GALACTOSIDASE | ***BGAL*** | **-** | **+** | **+** | **-** | **+** | **+** | **-** | **+** | **+** | **+** |
|  | L-PYRROLYDONYL-ARYLAMIDASE | ***PYRA*** | **-** | **+** | **+** | **-** | **-** | **-** | **-** | **+** | **-** | **-** |
|  | ALPHA-GALACTOSIDASE | ***AGAL*** | **-** | **+** | **+** | **-** | **+** | **+** | **-** |  | **+** | **+** |
|  | ALANINE ARYLAMIDASE | ***ALAA*** | **-** | **+** | **+** | **-** | **-** | **-** | **-** | **+** | **-** | **-** |
|  | TYROSINE ARYLAMIDASE | ***TYRA*** | **-** | **+** | **+** | **+** | **+** | **-** | **+** | **+** | **+** | **+** |
|  | ETA-N-ACETYL-GLUCOSAMINIDASE | ***BNAG*** | **+** | **-** | **+** | **-** | **-** | **-** | **-** | **-** | **-** | **-** |
|  | ALA-PHE-PRO ARYLAMIDASE | ***APPA*** | **-** | **+** | **+** | **-** | **-** | **-** | **-** | **+** | **-** | **-** |
|  | CYCLODEXTRIN | ***CDEX*** | **-** | **-** | **-** | **-** | **-** | **-** | **-** | **-** | **-** | **-** |
|  | ALPHA-MANNOSIDASE | ***AMAN*** | **-** | **-** | **+** | **-** | **-** | **-** | **-** | **+** | **-** | **-** |
|  | GLYCINE ARYLAMIDASE | ***GLYA*** | **-** | **-** | **-** | **+** | **+** | **-** | **+** | **-** | **-** | **+** |
|  | N-ACETYL-D-GLUCOSAMINE | ***NAG*** | **-** | **-** | **-** | **-** | **-** | **-** | **-** | **-** | **-** | **-** |
|  | BETA-GLUCOSIDASE | ***BGLU*** | **-** | **+** | **+** | **-** | **-** | **+** |  | **+** | **-** | **-** |
|  | BETA-MANNOSIDASE | ***BMAN*** | **-** | **+** | **+** | **-** | **-** | **-** | **-** | **+** | **-** | **-** |
|  | ALPHA-GLUCOSIDASE | ***AGLU*** | **-** | **+** | **+** | **-** | **-** | **-** | **-** | **+** | **-** | **-** |
| **ANTIBIOTIC RESISTANCE** | KANAMYCIN RESISTANCE | ***KAN*** | **-** | **-** | **-** | **+** | **-** | **-** | **-** | **-** | **-** | **-** |
|  | OLEANDOMYCIN RESISTANCE | ***OLD*** | **-** | **+** | **+** | **-** | **-** | **+** | **-** | **-** | **-** | **-** |
|  | POLYMIXIN_E RESISTANCE | ***POLYB_R*** | **-** | **-** | **-** | **-** | **-** | **-** | **-** | **-** | **-** | **-** |
| **OTHER** | METHYL-A-D-GLUCOPYRANOSIDE ACIDIFICATION | ***PHC*** | **+** | **-** | **-** | **+** | **+** | **+** | **+** | **-** | **+** | **+** |
|  | ELLMAN | ***MDG*** | **-** | **-** | **-** | **-** | **-** | **-** | **-** | **-** | **-** | **-** |
|  | PHOSPHORYL CHOLINE | ***ELLM*** | **+** | **+** | **+** | **+** | **+** | **-** | **+** | **+** |  | **+** |
|  | PUTRESCINE ASSIMILATION | ***PSCNA*** | **-** | **-** | **-** | **-** | **-** | **-** | **-** | **-** | **-** | **-** |
|  | GROWTH IN 6.5% NAC | ***NAC 6.5%*** | **-** | **-** | **+** | **-** | **-** | **-** | **+** | **-** | **+** | **-** |
|  | ESCULIN HYDROLYSIS | ***ESC*** | **-** | **+** | **+** | **+** | **-** | **+** | **+** | **-** | **+** | **+** |
|  | TETRAZOLIUM RED | ***TTZ*** | **-** | **-** | **-** | **-** | **-** | **-** | **-** | **-** | **-** | **-** |

|  | | **GN** | |
| --- | --- | --- | --- |
|  |  | **Genus** | *Pseudomonas* |
|  |  | **Species** | *fluorescens* |
|  |  | **Sample** | 24 |
| **SUGAR BREAKDOWN** | ADONITOL | ***ADO*** | **-** |
|  | L-ARABITOL | ***IARL*** | **-** |
|  | D-CELLOBIOSE | ***dCEL*** | **-** |
|  | D-GLUCOSE | ***dGLU*** | **-** |
|  | FERMENTATION/ GLUCOSE | ***OFF*** | **-** |
|  | D-MALTOSE | ***dMAL*** | **-** |
|  | D-MANNITOL | ***dMAN*** | **-** |
|  | D-MANNOSE | ***dMNE*** | **-** |
|  | D-SORBITOL | ***dSOR*** | **-** |
|  | SACCHAROSE/SUCROSE | ***SAC*** | **-** |
|  | D-TAGATOSE | ***dTAG*** | **-** |
|  | D-TREHALOSE | ***dTRE*** | **-** |
|  | MALONATE | ***MNT*** | **-** |
|  | 5-KETO-D-GLUCONATE | ***5KG*** | **-** |
| **ENZYMATIC ACTIVITY** | ALA-PHE-PRO-ARYLAMIDASE | ***APPA*** | **+** |
|  | L-PYRROLYDONYL-ARYLAMIDASE | ***PyrA*** | **-** |
|  | BETA-GALACTOSIDASE | ***BGAL*** | **-** |
|  | H2S PRODUCTION | ***H2S*** | **-** |
|  | BETA-N-ACETYL-GLUCOSAMINIDASE | ***BNAG*** | **-** |
|  | GLUTAMYL ARYLAMIDASE PNA | ***AGLTp*** | **-** |
|  | GAMMA-GLUTAMYL-TRANSFERASE | ***GGT*** | **-** |
|  | BETA-GLUCOSIDASE | ***BGLU*** | **-** |
|  | BETA-XYLOSIDASE | ***BXYL*** | **-** |
|  | BETA-ALANINE ARYLAMIDASE PNA | ***BAIap*** | **-** |
|  | L-PROLINE ARYLAMIDASE | ***ProA*** | **+** |
|  | LIPASE | ***LIP*** | **-** |
|  | PALATINOSE | ***PLE*** | **-** |
|  | TYROSINE ARYLAMIDASE | ***TyrA*** | **+** |
|  | UREASE | ***URE*** | **-** |
|  | ALPHA-GLUCOSIDASE | ***AGLU*** | **-** |
|  | BETA-N-ACETYL-GALACTOSAMINIDASE | ***NAGA*** | **-** |
|  | ALPHA-GALACTOSIDASE | ***AGAL*** | **-** |
|  | PHOSPHATASE | ***PHOS*** | **-** |
|  | GLYCINE ARYLAMIDASE | ***GlyA*** | **+** |
|  | ORNITHINE DECARBOXYLASE | ***ODC*** | **-** |
|  | LYSINE DECARBOXYLASE | ***LDC*** | **-** |
|  | DECARBOXYLASE BASE | ***0DEC*** | **-** |
|  | BETA-GLUCURONIDASE | ***BGUR*** | **-** |
|  | GLU-GLY-ARG-ARYLAMIDASE | ***GGAA*** | **-** |
| **ANTIBIOTIC RESISTANCE** | O/129 RESISTANCE (COMP.VIBRIO.) | ***O129R*** | **-** |
| **OTHER** | CITRATE (SODIUM) | ***CIT*** | **+** |
|  | L-LACTATE ALKALINIZATION | ***ILATk*** | **+** |
|  | SUCCINATE ALKALINIZATION | ***SUCT*** | **-** |
|  | L-HISTIDINE ASSIMILATION | ***IHISa*** | **+** |
|  | COUMARATE | ***CMT*** | **-** |
|  | L-MALATE ASSIMILATION | ***IMLTa*** | **+** |
